# Challenges, Potential Solutions in Recruiting and Retaining Participants for Alcohol Use Disorder Research: A Literature Review

**DOI:** 10.3390/healthcare14172826

**Published:** 2026-09-03

**Authors:** Jaskaran Lamba, Jonathan Shaw, Jason Zimmerman, Brian Kavin, Tara Menon, Charles Trevelyan, Shawn Koh

**Affiliations:** 1 School of Medicine, Meharry Medical College, Nashville, TN 37208, USA; jaskaran.lamba@mmc.edu; 2 School of Medicine, California University of Science and Medicine, Colton, CA 92324, USA; 3 Psychiatry, Eisenhower Health, Rancho Mirage, CA 92270, USA; 4 School of Medicine, Virginia Tech Carilion School of Medicine, Roanoke, VA 24016, USA; tarapm@vt.edu

**Keywords:** alcohol use disorder, participant recruitment, participant retention, attrition, research participation, clinical trial methodology, stigma, health disparities, narrative review

## Abstract

Objectives: This review aimed to identify the participant-, logistical-, and system-level factors that impede recruitment and retention in AUD research; to examine the strategies proposed to address them, including community-based and technology-assisted approaches; and to appraise the strength of the evidence supporting each strategy in order to define priorities for future work. Methods: A narrative review with thematic content analysis was conducted. PubMed/MEDLINE, PsycINFO, Scopus, Web of Science, CINAHL, and Embase were searched for records published between 1990 and 2026, supplemented by reference-list screening of included studies and relevant reviews. Records were eligible if they addressed recruitment, enrolment, retention, attrition, engagement, or follow-up in AUD or alcohol-related research. Given heterogeneity in design, population, and setting, findings were synthesized thematically rather than pooled statistically; a formal risk-of-bias assessment was not undertaken, although study design, sample characteristics, and author-reported limitations informed interpretation. Results: Sixteen challenges were identified across four domains: participant-level, access and logistics, study design, and system-level. Barriers were consistently better characterized than their remedies. The strategies with the most direct support were multimodal participant tracking, which sustained 81% retention at twelve months in an emergency department substance use cohort, and multidisciplinary or collaborative care structures, which produced the largest engagement differences observed. Conclusions: Attrition in AUD research is substantially a function of modifiable study design decisions rather than fixed participant characteristics. Priorities for future work include trials with research retention as a prespecified primary outcome, comparative evaluation of compensation structures, and standardized reporting of screening, enrolment, and attrition data.

## 1. Introduction

Alcohol use disorder (AUD) is the most prevalent substance use disorder worldwide [1]. The terminology regarding alcohol use and alcohol-related issues and disorders has evolved over time, ranging from unhealthy alcohol use [2] and risky use to harmful drinking [3]. It is characterized by a pattern of alcohol use leading to clinically significant distress as manifested by psychosocial, behavioral, and physiologic criteria [4]. The effects of this disorder are significant. Approximately 3 million deaths annually can be attributed to alcohol-related incidents [5] with a cost to society exceeding $250 billion annually [6]. This narrative review examines the challenges associated with recruiting and retaining participants in AUD research and evaluates current and emerging strategies to address them. Particular attention is given to digital recruitment approaches, including social media, as well as to the impact of eligibility criteria and cognitive functioning on participation across different stages of clinical trials.

Although the barriers to participation in AUD research have been cataloged repeatedly, the strategies proposed to overcome them have rarely been evaluated against recruitment or retention outcomes in AUD populations. Much of what is recommended is extrapolated from general clinical-trial methodology or from mixed substance use populations, and is reported as program description rather than as tested intervention. This review addresses that asymmetry by grading the evidence behind each proposed solution separately from the evidence establishing the barrier it is meant to solve. Accordingly, this review is organized around four questions. RQ1: Which participant-, logistical-, and system-level factors impede recruitment and retention in AUD research? RQ2: Which of these factors are properties of the population itself, and which are artifacts of study design? RQ3: What strategies have been proposed or tested to address them, and how strong is the evidence supporting each? RQ4: Where are the principal evidence gaps, and what study designs would close them? Together, these questions provide the framework for critically evaluating the current evidence on recruitment and retention in AUD research and identifying priorities for future investigation.

## 2. Methods

A narrative literature review and synthesis approach was used to examine the challenges associated with recruiting and retaining participants in alcohol use disorder (AUD) research and to identify potential strategies for addressing these challenges. A narrative approach was selected because the available literature encompasses diverse study designs, participant populations, research settings, and recruitment and retention strategies. This approach allowed findings from heterogeneous sources to be critically examined, compared, and synthesized thematically rather than requiring statistical pooling of results.

A systematic and comprehensive search of the published literature was conducted with the databases: PubMed/MEDLINE, PsycINFO, Scopus, Web of Science, CINAHL, and Embase. The search covered the period from 1990 to 2026. Additional relevant articles were identified by examining the reference lists of included studies and relevant review articles. Representative search terms included: “alcohol use disorder,” “alcohol dependence,” “alcohol misuse,” “alcohol use,” “clinical research,” “research participants,” “participant recruitment,” “recruitment,” “participant retention,” “retention,” “attrition,” “dropout,” “engagement,” “study participation,” and “participant follow-up.”

Studies were considered eligible if they addressed participant recruitment, enrollment, retention, attrition, engagement, or follow-up in the context of AUD or alcohol-related research. Records were excluded if they were unavailable in English; if they addressed substance use disorders in aggregate without disaggregating alcohol-related findings; if they reported recruitment or retention solely for clinical service delivery with no transferable implication for research conduct; or if they were conference abstracts, editorials, or commentaries without primary data or substantive synthesis. Where multiple reports described the same cohort, the most complete report was retained.

This review utilized the Scale for the Assessment of Narrative Review Articles to ensure a high quality narrative review [7]. Analysis proceeded inductively. Each included record was read in full; passages bearing on recruitment, enrollment, retention, or attrition were extracted verbatim into a shared matrix; extracts were assigned descriptive codes; and codes were grouped into higher-order themes through iterative constant comparison. The narrative synthesis focused on identifying recurring patterns and relationships across the literature. Emerging themes were identified and summarized in the following section. Because this review used a narrative synthesis approach and sought to provide a broad overview of the literature, a formal risk-of-bias assessment was not undertaken. However, study design, sample characteristics, and limitations reported by the original authors were considered when interpreting and synthesizing the findings.

## 3. Challenges in Alcohol Use Disorder Research

**Figure 1:**
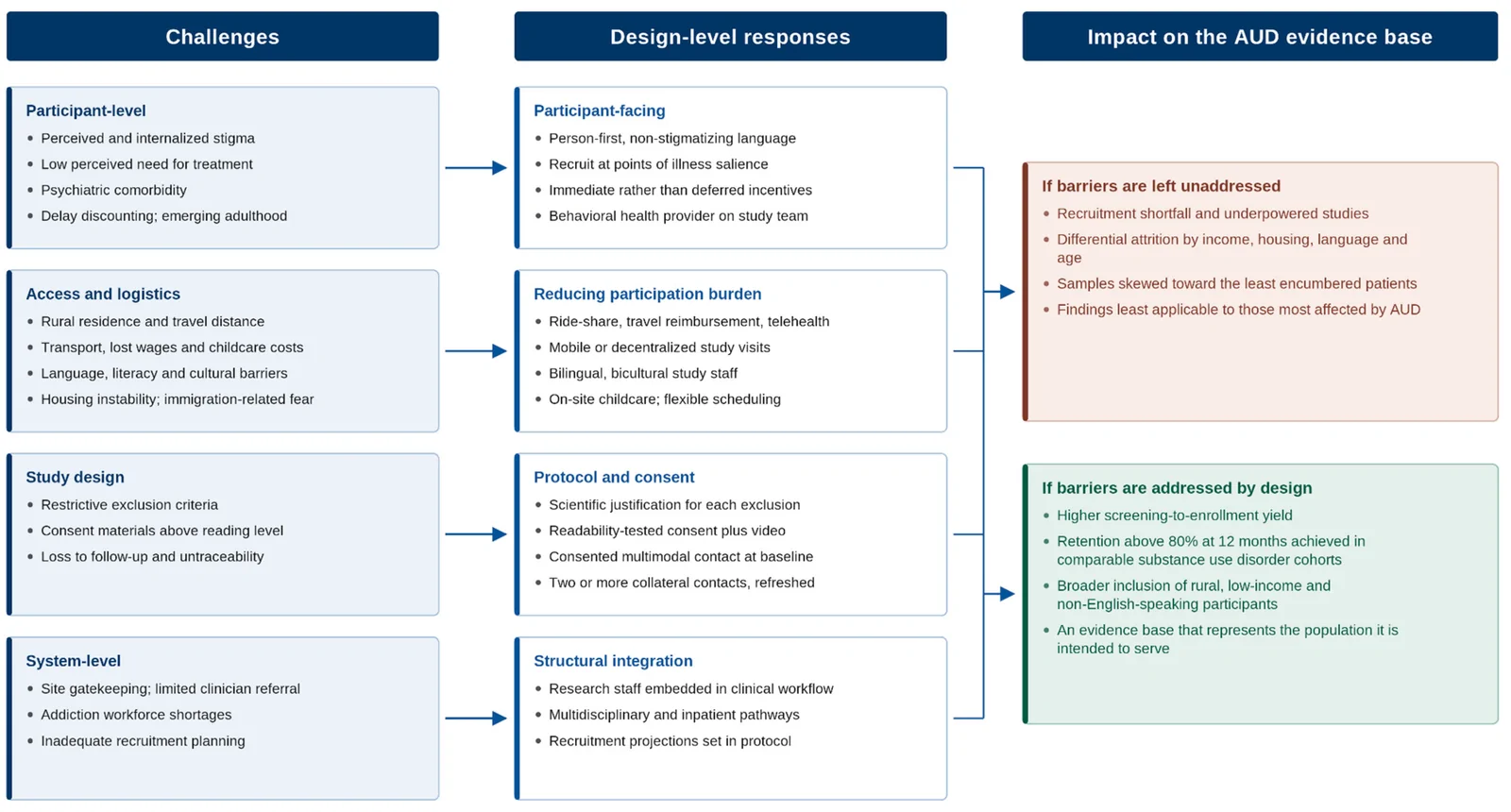
Conceptual model of recruitment and retention in alcohol use disorder research.

Research on AUD faces numerous challenges that impede effective recruitment, retention, and understanding of the disorder. Barriers to researching AUD are multifaceted, encompassing personal, social, and systemic factors that might hinder access and engagement [8]. This section groups them into four domains. Participant-level factors describe characteristics and experiences of individuals with AUD that shape their willingness and ability to engage with research, including stigma, insight into the disorder, psychiatric comorbidity, delay discounting, and developmental stage. Access and logistical barriers describe the practical circumstances under which participation is attempted, including geography, cost, caregiving responsibilities, cultural and linguistic concordance, housing and employment stability, and the broader sociopolitical climate. Study design factors describe features of research protocols themselves that determine whether enrolled participants can be retained, including eligibility criteria, follow-up procedures, and the accessibility of consent materials. System-level factors describe the institutional and workforce context in which AUD research is conducted, including clinical gatekeeping, workforce capacity, and the degree to which recruitment is embedded within routine clinical care. Corresponding strategies for each domain are addressed in Section 3.4, and the full mapping of challenges to proposed solutions with supporting levels of evidence is presented in Table 1 and Figure 1.

### 3.1. Participant-Related Challenges

#### 3.1.1. Stigma and Shame

Stigma is among the most consistently identified barriers to engaging with AUD care, and it operates through two distinct mechanisms with different implications for research participation. Perceived stigma reflects an individual’s self-beliefs about how others view people with AUD, while self-stigma reflects the internalization of those beliefs into one’s own self-concept. In a study of college students, Jennings and colleagues found that both forms of stigma independently impede help-seeking, and that perceived stigma appears to increase self-stigma over time, shifting individuals toward a more self-reliant approach to reducing their drinking rather than pursuing formal care [9]. Although this work was conducted in a treatment-seeking context, the mechanisms translate to research recruitment. Enrolling in a study requires disclosing one’s drinking to research staff, signing formal consent documents, and accepting that data will be recorded and stored—a set of disclosure demands that may exceed those of a clinical encounter, particularly for participants with high self-stigma. Consistent with this, a cross-sectional study of the general Danish population found that perceived barriers to seeking help for AUD were closely tied to shame and disclosure concerns, and were more pronounced among individuals with more severe alcohol abuse [10]. The framing used to describe AUD may itself reinforce these barriers. Shi et al. have argued that continued use of pejorative or moralizing terminology in clinical research materials sustains public stigma, discourages engagement, and shapes how individuals with AUD understand their own condition [11]. Together, these findings suggest that recruitment materials, consistent processes, and study-staff communication should be reviewed with stigma in mind—both in the language they use and in the disclosure demands they place on prospective participants.

#### 3.1.2. Individual Perception and Misclassification

Another barrier to recruitment is an individual’s insight into whether they believe they need treatment at all [50]. Many individuals with AUD believe that they can either handle it themselves or that their AUD is not bad enough to require treatment. In a series of three national alcohol use surveys among Latinos in the United States, 61.2% of participants reported not needing treatment at all [12].

A study exploring the lived experiences of alcohol use disorder patients in Rwanda identified similar themes and found that recognition of the need for care was often triggered by a crisis [51]. Participants frequently sought medical help only after alcohol-related consequences became severe or unmanageable, including acute psychological distress, behavioral dysregulation, and serious medical complications [51]. These crisis points appeared to function as turning points that prompted individuals to recognize the need for professional support, sometimes following intervention from concerned family members [51]. This suggests that periods of acute illness or crisis may represent important opportunities for engaging individuals in both treatment and research [51].

A recruitment log analysis from the TREAT consortium’s alcoholic hepatitis studies illustrates how a participant’s perceptions may play out at the point of enrollment. Among patients who declined to participate and provided a reason, the most commonly cited included feeling too sick to take part, a desire to concentrate on abstinence rather than take on the added demands of research, and a lack of interest in research participation despite available financial or tangible incentives [13]. A subset of patients declined on the grounds that they did not believe their drinking was problematic in the first place, echoing the low perceived need for treatment described earlier. Shi et al. observed that the use of pejorative terms can create negative biases that discourage an individual’s willingness to seek treatment and also impact the broader societal understanding of this disorder [11].

#### 3.1.3. Comorbid Psychiatric Disorders

Studies have shown that individuals with AUD often experience high rates of co- occurring mental health issues, further complicating diagnosis and treatment [16]. The interplay between alcohol dependence and internalizing disorders suggests a bidirectional relationship that complicates treatment approaches [15]. AUD is often complicated by comorbid mental health conditions. Castillo–Carniglia et al. examined the main psychiatric disorders associated with AUD [14]. They demonstrated that the lifetime prevalence of AUD was associated with a wide range of psychiatric disorders, including antisocial personality disorder (77%), borderline personality disorder (52%), and major depressive disorder (27% to 49%). These comorbidities can further complicate treatment initiation and adherence. Major depressive disorder alone can increase an individual’s self-stigma, resulting in the decision to avoid treatment [17]. Reduced feelings of self-worth associated with major depressive disorder can lead to an individual believing they are unworthy of treatment.

#### 3.1.4. Devaluing Future Rewards

Time discounting or the tendency to devalue future rewards, compared to immediate rewards, is prevalent among individuals with AUD [20], a tendency that may have a neurobiological basis, as chronic alcohol exposure has been shown in animal models to impair reward-related decision-making circuitry in the striatum [52]. This tendency may affect an individual’s engagement with long-term research projects. Among 84 substance-dependent individuals in an inpatient detoxification program, delayed discounting at treatment entry predicted shorter treatment retention and higher odds of premature dropout. This effect was partially mediated by lower treatment readiness [21]. Since participation in research can require sustained engagement for future benefits (a cash reward upon completion and potential treatment access), it may be reasonable to infer that individuals with steep delay discounting may find it difficult to maintain commitment to multi-visit longitudinal studies where rewards are deferred. Additionally, the behavioral economic framework may further support this inference.

#### 3.1.5. Geographic Barriers

Geographic and logistical challenges can create unique barriers to AUD treatment recruitment, especially for those who live in rural settings. Individuals in rural settings, women in particular, are significantly less likely to receive treatment for AUD [25]. Individuals in rural communities often face additional stigma, since such communities tend to be small, where knowledge of one seeking help for AUD may be more likely to spread around town.

#### 3.1.6. Financial Barriers

Cost presents a barrier to both AUD treatment and participation in AUD research. Federal law now requires insurers to cover SUD treatment under the Mental Health Parity and Addiction Equity Act (MHPAEA), but federal reporting through 2024 found inadequate compliance [53]. In May 2025, enforcement of the MHPAEA final rule was paused entirely [54]. In practice, a cross-sectional study of 1053 treatment-naive adults that investigated barriers to AUD treatment found that 7.9% of respondents who were receptive to treatment had insurance that did not cover the cost [27]. Additionally, 14.2% of respondents reported being unable to cover the expense of treatment [27]. Subgroup analysis found that those with high barriers overall cited financial barriers more frequently. Overall, a 2019 systematic review concluded that economic differences proved an impactful barrier to treatment [50]. There is substantial overlap between barriers to treatment and barriers in research recruitment, but the financial barriers specific to research participation deserve separate attention.

Even individuals motivated to enroll in a study can be deterred by costs. Transportation, parking, missed wages, and childcare expenses can accumulate with each study visit. In a qualitative study of patients with comorbid AUD and alcohol-related liver disease enrolled in a UK clinical trial, participants cited transportation expenses and the inability to miss paid work as reasons they could not reliably attend study visits [8]. These costs fall disproportionately on individuals with unstable housing or employment who are overrepresented among those with AUD, driving attrition among enrolled participants even when initial recruitment succeeds [55].

#### 3.1.7. Cultural and Gender-Specific Barriers

Compared to Caucasians, ethnic minorities were half as likely to receive treatment for AUD by primary care providers or specialists [30]. The barriers to treatment are multifactorial, but some major contributors to the disparities in care are hypothesized to result from linguistic barriers, access to primary care, and knowledge of treatment interventions [12]. Disparities have also been demonstrated between genders. Despite increasing rates in AUD diagnosis in women, women are significantly less likely to receive treatment [29]. The explanation behind these barriers results from both extrinsic and intrinsic factors, including income, access to childcare, the societal stigma of being labeled a bad mother, and fear of involvement by child protective services. Differences in cultural backgrounds between researchers and participants may lead to differing perspectives and misunderstandings. This has the potential to influence success in recruiting and retaining study populations in question [56].

#### 3.1.8. Socioeconomic Factors

Lee et al. found that individuals with AUD in lower income brackets have unstable employment, and therefore often lack stable housing. These socioeconomic challenges negatively impact their quality of life and can pose barriers to consistent participation in research [32]. Lee and colleagues further examined how homelessness is associated with a higher prevalence of substance use disorders. Lack of stable housing and other social determinants of health can impede access to treatment and participation in research studies [56]. White non-Hispanic Americans have also been shown to be subject to what has been termed, the “depths of despair,” resulting in premature deaths from suicide, poisoning, and alcohol-related deaths. This despair might arise from trauma of various sorts, including unresolved grief and posttraumatic stress disorder, under- or unemployment, and having a social disorder [57]. A recent qualitative study of hospitalized Latinx men with AUD reinforces these disparities from the patient’s own perspective: participants described social factors such as housing instability [33]. Two participants experiencing homelessness described being unable to prioritize AUD goals when faced with the prospect of living on the streets [33]. One participant stated “The first thing I need here where I am, which I’ll repeat, is only a temporary place, a housing assessment, and to apply. Because I need a stable place and to stay in one location, not be in the streets.” [33].

#### 3.1.9. Emerging Adulthood

Emerging adults (ages 18–25) represent a group with both the highest prevalence of SUDs (in the USA and Canada) and a higher rate of dropout from SUD studies compared to adults 26+. Factors that may contribute to low retention include lower abstinence motivation, lower readiness to change, higher psychiatric comorbidity, and ongoing identity exploration relative to older adults [23].

### 3.2. System-Level and Sociopolitical Obstacles

Research environment and funding constraints further complicate recruitment and retention efforts. Watson et al. observed that recruitment difficulties are a long-standing issue in randomized controlled trials, often leading to publication bias due to under-recruitment [47]. Time constraints and inadequate remuneration can also hinder participant retention, ultimately affecting the statistical power of trial results [58]. Moreover, the need for early and thorough planning in recruitment strategies with remuneration has been recommended [48,49]. Given that immigrants make up over 13% of the US population and 10.7 million of them are undocumented, it is worth considering how being part of this demographic impacts care-seeking behaviors or the desire to participate in medical research studies [59,60]. One study examined healthcare providers’ perspectives on the impact of immigration and customs enforcement on patients’ mental health and found that heightened immigration enforcement in communities led to increased fear of deportation, not only among undocumented immigrants, but also among their US citizen children and networks of families and friends. This pervasive fear led to health care avoidance, stress and anxiety, all of which have profound effects on physical and mental health [34]. Acute stress can occasionally lead individuals to choose alcohol even when they prefer the non-alcoholic alternative [61]. Although previous studies showed stress increases alcohol’s absolute value, alcohol’s value relative to alternatives may be more relevant for drinking decisions.

### 3.3. Logistical Challenges

Difficulties accessing populations indicate that, at times, practice managers or administrators can act as gatekeepers, potentially impeding recruitment efforts in primary care settings [42]. This aligns with findings from Thørrisen et al., who noted that internal organizational barriers, such as limited time and resources, are significant impediments to implementing alcohol prevention programs in occupational health services [43]. Paradise et al. noted that workforce shortages across roles and settings have limited the ability of the system to meet the demand for treatment in addiction care [44].

Two systemic factors, the burden placed on study coordinators to identify eligible patients and the degree of active clinician involvement and recruitment, appear particularly influential across the literature. When TREAT Consortium research coordinators were surveyed about what would make their alcoholic hepatitis studies more efficient, the two most common responses were less time spent searching for patients to enroll and greater physician involvement in recruiting [13]. This pattern is consistent with broader observations about gatekeeping and workforce capacity in addiction research. Balio et al. found that practice managers and administrators frequently act as gatekeepers to primary care populations, impeding recruitment even when a study is otherwise well-designed [42]. Thørrisen et al. similarly noted that the internal organizational barriers, particularly limited time and resources, obstruct the implementation of alcohol-related programs in occupational health settings [43]. At a broader level, Paradise and Wakeman have documented that workforce shortages across roles and settings have limited the capacity of addiction care system to meet even routine clinical demand, let alone accommodate the additional workload of research recruitment [44]. Taken together, these findings suggest that recruitment yield in AUD research is shaped less by any single institutional failure than by a set of interacting workforce and workflow constraints that concentrate the burden on already strained study staff.

### 3.4. Recruitment and Retention Solutions

#### 3.4.1. Methodologic Adjustments for Clinical Studies

Treatment seekers with AUD differ from nontreatment seekers in various domains, including the pattern of alcohol use, age, AUD severity, and degree of impulsivity and liver function [38,39,40,41]. In treatment-seeking populations, alcohol can be provided under ethically acceptable conditions without placing patients at a disadvantage [62,63,64,65]. The wider inclusion of treatment-seeking individuals in clinical trials can be helpful in increasing the external validity of clinical studies [44,66]. Even well-resourced, multifactorial recruitment frameworks developed for minority and low-SES populations have historically excluded individuals with active substance use from enrollment, underscoring that general health-disparities recruitment models cannot be applied to AUD research without modification for this population’s specific presentation and substance use habits.

Study design itself can be adapted to reduce the geographic and logistical barriers described earlier in this review. In the HANDLS study, a 20-year longitudinal investigation of health disparities in a racially and socioeconomically diverse Baltimore cohort, researchers deployed a mobile medical research vehicle to bring study visits directly into participants’ neighborhoods, alongside a multilevel recruitment and retention strategy informed by the prior literature, community stakeholders, and a standing community advisory board [26]. This approach directly targeted the transportation and rural-access barriers noted in Section 3.1.5, and offers a transferable model for AUD researchers seeking to enroll populations for whom travel to a fixed clinical site is itself a barrier to participation. Of note, recruitment and retention strategies for disadvantaged and marginalized groups may be different across respective populations. Metayer et al. demonstrated that media announcements were more effective than collaborations with community organizations in facilitating the recruitment of Haitian families [31].

#### 3.4.2. Multidisciplinary Approach/Collaboration

For patients with AUD and liver disease, where abstinence is a crucial therapeutic goal, treatment teams should consider including a hepatology specialist [66]. This approach has been successful in practice. One multidisciplinary alcohol-associated liver disease clinic established in 2018 combined a hepatologist, psychiatrist, psychologist, nurse, and social worker under one shared referral pathway. An initial cohort was followed for six months with no requirement for abstinence and 75% were still active participants at completion. The clinic demonstrated feasibility and promising early engagement outcomes, providing a model that could better serve to facilitate research recruitment than through single specialty alone [45]. More broadly, a 2023 retrospective cohort study found that patients supported by a multidisciplinary team had 6-month visit-continuation rates of 91.7% compared to 38.7% among those without one. This gap persisted at 12 months (90.6% vs. 25.8%) [46]. Although these studies measured treatment continuation rather than research retention, the magnitude of the effect suggests that a team-based, multidisciplinary structure could meaningfully reduce attrition in AUD research.

The benefit of collaborative care has also been shown in the primary care setting. The SUMMIT randomized clinical trial for AUD and opioid use disorder found that adding a behavioral health provider to the primary care team increased both treatment initiation (31.6% vs. 13.7%), defined as a second visit after identification, and engagement (15.5% vs. 4.2%) relative to usual care [18]. This again suggests that both recruitment and retention could be increased by integrating a collaborative care model into research protocols. Additionally, a secondary analysis of the SUMMIT trial found that initiation was the primary driver of improved abstinence, pointing to the first follow-up contact as a superior opportunity for enrolling a patient into a study [19].

#### 3.4.3. Recruitment, Tracking, and Follow-Up

White and Hind explored various modes of recruiting patients to primary care research [48]. After having examined recruitment rates, planning techniques, and influences on poor estimation, they determined that early planning of the recruitment process was essential to improving trial timelines. Tracking and follow-up with patients posed further complications since confidentiality must be seriously considered. Furthermore, it is essential to collect pertinent contact information and keep it current, which is sometimes difficult in this population, owing in part to the tendency to transiency, mental illness, and homelessness. McKenzie and colleagues recommend organized advanced planning [35,67]. Unfortunately, losing study participants to follow-up can lead to bias or prolongation of the study. McKenzie et al., further examining the issue based on previous studies, recommended paying attention to staff training and support, establishing rapport with participants, assuring them of confidentiality, and using agency and field tracking with particular attention to staff safety issue with the hopes of increasing recontact rates to 90% of study cohorts [36,68,69,70,71,72,73,74,75]. Worth et al. found that clinical trial recruitment and retention were even more challenging in the ED setting [37]. They sought individuals in this setting, seeking to enhance the 12-month retention rates. Tracking and retention occurred over a 29-month period. They contacted participants via phone messages (the most common method), text messaging, emails, online searches, incarceration websites, home visits, and private messages on Facebook pending approval by the local IRB. This process resulted in 3-, 6-, and 12-month retention rates of 89%, 86%, and 81%, respectively. These rates were obtained in the SMART-ED trial, a six-site study conducted in urban academic emergency departments, and several features of that study limit their transferability to AUD research. Eligibility required a positive screen for problematic use of a non-alcohol, non-nicotine drug, so alcohol was excluded as the index substance, and the most common primary substances were cannabis (44%), cocaine (27%), and street opioids (17%). Enrollment further required adequate English proficiency, access to a telephone, and the ability to name at least two reliable locators, while the protocol excluded individuals with significant cognitive impairment, those in police custody, those already engaged in addiction treatment, and those residing more than 50 miles from the follow-up site. Retention was supported by dedicated follow-up staff who made an average of 26 contact attempts per participant, by compensation of $50 at baseline and $75 at each follow-up visit, and by private messaging through social media at those sites whose institutional review boards permitted it. The authors also note that widening the follow-up window from four to six weeks may have inflated the reported rates slightly. These figures are therefore best read as an indication of what is achievable with intensive, well-resourced tracking in an acute care setting, and as support for the use of multiple contact methods, rather than as a retention benchmark for AUD research specifically. In the COMPEERS study: A Cluster Randomized Feasibility Study of Contingency Management and PEER Support to Promote Attendance, Increase Treatment Engagement and Improve Outcomes for People with Dual Diagnoses of Psychosis and Drug/Alcohol Problems, attendance was incentivized using Contingency Management (CM) principles, a behavioral strategy that relies on a consistent reward schedule to reinforce learning. Because standard clinical visits are too infrequent, CM requires closer monitoring so that participants can be rewarded regularly. Delivering a financial reward such as a £10 shopping voucher immediately after the participant achieves the target behavior is essential, as it may create a strong psychological link between their action and the subsequent payoff [76].

Beyond contact and tracking, remote visit modalities may reduce attrition by eliminating the transportation and scheduling barriers that drive dropout. A 2025 systematic review of 34 RCTs found that remote interventions supplementing in-person care reduced relapse rate by 39%, while a 2023 retrospective study found early telehealth via video was associated with lower dropout hazard (HR 0.64) and greater odds of sustained engagement (OR 5.40) [77,78]. These findings suggest that substituting telehealth for non-essential in-person study visits could improve retention, particularly for participants facing the geographic and financial barriers described in Section 3.1.5 and Section 3.1.6.

Additional strategies included consent for contact through social media, the location of the study site, and the use of the following exclusion criteria: significant cognitive impairment or judgment, being in police custody at the time of treatment, current engagement in addiction treatment, residing more than 50 miles from the location of follow-up visits, inability to provide sufficient contact information, and prior participation in the current study. Hospitalization itself may function as a recruitment opportunity rather than only a treatment setting. In a qualitative study of Latinx men with AUD followed after discharge, participants described hospitalization as an actionable moment for change with one participant stating “*Not drinking is definite. I can’t drink. My liver is bad, my kidneys are bad, and my stomach is bad. It’s time to stop.* Another participant of the same study described how being hospitalized pushed him to ponder his own mortality and how the continuation of drinking could affect his family. He stated “*I don’t want to die. I want to be with my kids.*” suggesting that inpatient addiction consult team models that actively integrate hepatology, primary care and psychological support into AUD treatment could also serve as a natural point of contact for enrolling patients into longitudinal research, particularly for populations such as Latinx men who possibly are underrepresented in AUD studies [33]. Culturally tailored recruitment strategies have also been shown to meaningfully affect retention outcomes in Latino populations specifically. In an evaluation of a community-based Latino family program, three strategies stood out as more effective than the others tested: in-person recruitment conducted by a trusted community liaison, facilitators who were bilingual, bicultural, and experienced, and the provision of free on-site child care [79]. These findings echo McKenzie et al.’s emphasis on staff training and rapport-building as retention drivers, and may also apply to other populations.

The study’s relationship to community institutions can itself function as a recruitment strategy. Partnering with institutions such as churches or free clinics, while also offering participants a tangible secondary benefit, for example, referrals to private practice physicians, community health centers, or clinics willing to treat underinsured or uninsured participants may improve recruitment beyond what either strategy accomplishes alone. In the HANDLS study, providing direct benefits to participants, including comprehensive health screenings and medical evaluations, was described by the investigators as an essential facet of successfully recruiting and retaining a cohort of 3722 minority and low-income participants. This finding is not unique to HANDLS: a Cochrane qualitative evidence synthesis of participants’ experiences across randomized trials similarly found that access to health checks or medications participants could not otherwise afford functioned as a meaningful factor sustaining engagement, distinct from and in addition to financial reimbursement. Taken together, these findings suggest that AUD researchers designing recruitment strategies around institutional partnerships should consider building in a genuine health-related benefit to participation, rather than relying on institutional access or compensation alone.

#### 3.4.4. Addressing Understanding, Financial Compensation and Counseling

Comprehension of materials is also a relevant factor for recruitment and retention. In the HANDLS study, all written materials were analyzed for readability using the Flesch–Kincaid Readability Scale, and a video explaining the study rationale was incorporated into the informed consent process. Given that individuals with AUD who come from lower socioeconomic backgrounds may face compounding and intersectional barriers related to health literacy, stigma, or distrust of medical research [56], readability-tested consent materials and short explanatory videos, could improve both initial comprehension at enrollment and participants’ confidence in remaining engaged through follow-up [80].

#### 3.4.5. Addressing Financial Burden and Compensation

Several approaches show promise for reducing costs for participants, though most of the evidence comes from substance-use or general clinical research populations rather than AUD-specific studies, and should be taken as suggestive rather than confirmed for this population. Multi-site cohort studies have identified advance planning for participant reimbursement as a key facilitator of recruitment and retention among underrepresented populations [55]. Fair, non-coercive compensation for participants’ time is similarly framed as a matter of research equity and has been associated with reduced attrition in SUD research [56,81,82]. Telehealth visits and rideshare-based transportation offer practical ways to reduce travel and time-related burden. One study found that participants provided with rideshare opportunities were retained at a rate above 95% [28,83]. Additional approaches like having financial counseling included in AUD research could help offset financial pressures.

#### 3.4.6. Retention Rates for Interventions in Emerging Adults

Dalton et al. examined which intervention types best sustain engagement for emerging adults (3.1.9). The authors reported on nine studies and found that CBT and contingency management alone significantly increased retention for alcohol and cannabis use disorders in this age group, however, further study was encouraged given the limited evidence base [23]. Contingency management, which provides tangible, near-term reinforcement for desired behaviors, may be particularly useful for research retention given the diminished future-reward orientation common both at this developmental stage, and among individuals with AUD (as seen in Section 3.1.4). A systematic review by Scholten et al. identified future-oriented manipulations and mindfulness-based approaches as the most promising behavioral strategies for reducing DD [24].

#### 3.4.7. Leveraging Social Determinants of Health (SDOH) Software

Modern SDOH platforms (e.g., Unite Us, Aunt Bertha/Socially Determined) are increasingly integrated into electronic health record systems and allow real-time identification of barriers matched to individual patient needs. In the context of AUD research, these platforms could be repurposed to generate individualized barrier-reduction plans based on SDOH screening results. For example, a participant flagged for transportation insecurity could be automatically be offered a ride-share program or offered telehealth visit alternatives, while a participant flagged for food insecurity could receive an immediate incentive related to food assistance counteracting delay discounting by making the reward for engagement immediate rather than delayed. With patient consent, researchers can deploy multi-channel online advertising and social media campaigns to reach specific demographic or geographic populations at elevated risk for both AUD and social vulnerabilities. Because utilizing multiple promotional activities is proven to improve overall trial recruitment, social media and targeted online advertising could serve as a highly effective “funnel” strategy to engage hard-to-reach individuals [84].

## 4. Limitations

This review has multiple limitations. First, the included studies were heterogeneous in design, population, setting, and outcome, and many of the strategies identified were supported by indirect evidence from treatment engagement or mixed substance-use populations rather than AUD-specific research participation. Second, a formal risk-of-bias assessment was not undertaken and thus limits the ability of the authors to quantify the potential for bias across the included evidence. Lastly, most of the studies included in this review are focused on western populations. Further exploration is needed on diverse populations that span participants with AUD from around the world. The narrative synthesis and interpretation may therefore be influenced by publication, selection, and reporting biases within the underlying literature and by the authors’ interpretation of the findings. These limitations should certainly be considered when interpreting the strength and generalizability of the conclusions made in this review.

## 5. Conclusions

Recruitment and retention remain the principal practical constraints on alcohol use disorder research, and the evidence reviewed here indicates that both respond more to study design than is commonly assumed. Three implications follow for investigators. Retention should first be treated as a design parameter and planned at the protocol stage rather than addressed after the first missed visit, since reimbursement, transportation, childcare, and the staffing required for participant tracking are budget decisions that shape attrition before the first participant is enrolled [85]. Second, the approaches associated with the highest retention in this review are those that reduce what participation costs the participant and that move recruitment closer to where patients already are, including consented multimodal contact (telephone, text message, email, social media, and field tracking), transportation assistance or telehealth in place of non-essential in-person visits, and recruitment embedded within primary care, inpatient, and multidisciplinary pathways rather than within a dedicated research site alone. These warrant priority where funding for retention is limited. Third, consent documents and study communications should be written for the population being enrolled: person-first and non-stigmatizing in language [86], tested for accessibility across a range of literacy and educational backgrounds, and delivered in the participant’s preferred language. For studies conducted in the United States, investigators should anticipate that concerns regarding immigration status may deter participation and should avoid collecting such information where it is not scientifically necessary. Eligibility criteria deserve the same scrutiny, particularly those excluding individuals with impaired cognition or executive function, non-treatment-seekers, and those with psychiatric comorbidity, as each exclusion narrows the applicability of the resulting evidence and should be justified rather than applied by default.

Most of the strategies described above have been evaluated against treatment engagement rather than against research participation, and six priorities for future work follow from this. These are (1) trials in which recruitment and retention are prespecified primary outcomes rather than quantities reported post hoc; (2) direct evaluation in AUD samples of strategies currently supported only by indirect evidence, including multidisciplinary and collaborative care models, telehealth substitution for in-person visits, rideshare-based transportation, and contingency management; (3) comparative evaluation of compensation structures, addressing amount, timing, and payment schedule, and testing whether front-loaded or visit-contingent payment reduces attrition without compromising voluntariness; (4) experimental evaluation of eligibility criteria themselves, quantifying the effect on retention and on external validity of enrolling non-treatment-seeking participants and those with cognitive impairment or psychiatric comorbidity; (5) standardized reporting of screening, eligibility, enrollment, reasons for refusal, and attrition, in sufficient detail to permit comparison across studies; and (6) studies conducted outside high-income Western settings and within groups underrepresented in the present literature, including women, rural residents, emerging adults, and ethnic and linguistic minority populations. Until these data exist, recruitment planning in AUD research will continue to depend on evidence borrowed from adjacent fields and applied by inference.

## Figures and Tables

**Table 1:** Recruitment and retention challenges in alcohol use disorder research, proposed solutions, and supporting level of evidence.

Domain	Identified Challenge	Key Supporting Evidence	Proposed Solution(s)	Level of Evidence
Participant-level	Perceived and internalized stigma; shame about disclosure	Jennings et al. [9] (three-path model, cross-sectional): perceived stigma and self-stigma both impede help-seeking and shift individuals toward self-reliance. Finn et al. [10] (cross-sectional, general population). Shi et al. [11]: pejorative terminology reinforces bias.	Person-first, non-stigmatizing recruitment language; explicit confidentiality assurance at first contact and at each visit; recruit via trusted community liaisons rather than clinical gatekeepers alone.	Barrier: IV Solution: V
Participant-level	Low perceived need for treatment or research participation	Zemore et al. [12]: 61.2% of Latino respondents across three national alcohol surveys reported no perceived need for treatment. TREAT Consortium recruitment-log analysis [13]: most common decline reasons were feeling too sick, wishing to focus on abstinence, and disbelief that drinking was problematic.	Frame study participation independently of treatment-seeking; recruit at moments of illness salience (hospitalization, ED contact); pair recruitment with public education on AUD as a treatable disorder.	Barrier: IV Solution: IV–V
Participant-level	Psychiatric comorbidity complicating eligibility, adherence and retention	Castillo-Carniglia et al. [14]: lifetime AUD associated with antisocial personality disorder (77%), borderline personality disorder (52%) and major depressive disorder (27–49%). Kushner et al. [15]; Kaufmann et al. [16]. Alqahtani and Pringle [17]: depression-related self-stigma deters care-seeking.	Integrate a behavioral health provider into the study team (SUMMIT RCT [18,19]: treatment initiation 31.6% vs. 13.7%; engagement 15.5% vs. 4.2%); justify rather than default to psychiatric exclusion criteria.	Barrier: III–IV Solution: II †
Participant-level	Delay discounting—devaluation of deferred rewards	Bailey et al. [20]. Inpatient detoxification cohort [21] (*n* = 84): baseline delay discounting predicted shorter retention and higher odds of premature dropout, partially mediated by lower treatment readiness.	Contingency management and front-loaded or immediate incentives (Pfund et al. [22] meta-analysis; Dalton et al. [23] systematic review); brief, episodic future-thinking and mindfulness exercises at study visits (Scholten et al. [24] systematic review).	Barrier: III CM: I † EFT/mindfulness: I (indirect) †
Participant-level	Emerging adulthood (ages 18–25): highest SUD prevalence, highest dropout	Dalton et al. [23] systematic review (9 studies): emerging adults show higher attrition than adults ≥ 26, attributed to lower abstinence motivation and readiness to change, higher psychiatric comorbidity and ongoing identity exploration.	Deliver retention-relevant components via CBT or contingency management, the only modalities shown to increase retention in this age band significantly; age-tailored contact modalities and scheduling.	I (limited/low-certainty evidence base)
Access and logistics	Geographic distance and rural residence	Davis and O’Neill [25]: rural residents, women in particular, are significantly less likely to receive AUD treatment; small-community visibility amplifies stigma.	Mobile medical research vehicle delivering visits within participants’ neighborhoods (HANDLS [26], *n* = 3722, 20-year cohort); telehealth assessment; satellite or decentralized visit sites.	Barrier: IV Solution: III †
Access and logistics	Direct and indirect costs of participation	Cross-sectional survey of 1053 treatment-naive adults [27]: 7.9% had insurance not covering treatment, 14.2% could not meet the expense. UK qualitative study of trial participants with AUD and alcohol-related liver disease [8]: transport costs and inability to miss paid work cited as reasons for missed visits.	Budget participant reimbursement at protocol stage; fair, non-coercive compensation; rideshare-based transport (>95% retention in one study [28]); telehealth substitution for non-essential visits; on-site financial counseling.	Barrier: IV Rideshare: IV † Counseling: V
Access and logistics	Caregiving responsibilities and gender-specific barriers	McCrady et al. [29]: despite rising diagnosis rates, women are significantly less likely to receive treatment; contributors include childcare access, stigma of being labeled an unfit mother, and fear of child protective services involvement.	Free on-site childcare during study visits; evening and weekend scheduling; plain-language written statement of mandatory-reporting boundaries during consent.	Barrier: IV Childcare: III–IV † Other: V
Access and logistics	Cultural and linguistic barriers; ethnic disparities in access	Mulia et al. [30]: ethnic minorities approximately half as likely as White patients to receive AUD treatment from primary care or specialists; contributors include language, primary care access and treatment awareness (Zemore et al. [12]).	Bilingual, bicultural and experienced study facilitators; in-person recruitment by a trusted community liaison; population-specific channel selection (Metayer et al. [31]: media announcements outperformed community-organization partnerships for Haitian families).	Barrier: IV Solution: III–IV †
Access and logistics	Housing and employment instability	Lee et al. [32]: unstable employment and housing among lower-income individuals with AUD impair quality of life and consistent participation. Qualitative study of hospitalized Latinx men [33]: participants described being unable to prioritize AUD goals while unhoused.	SDOH screening linked to referral platforms generating individualized barrier-reduction plans; agency and field tracking; provision of tangible health benefits alongside reimbursement (HANDLS [26]; Cochrane qualitative evidence synthesis).	Barrier: IV SDOH platforms: V Health benefits: III + I (qual.)
Access and logistics	Sociopolitical climate and immigration-related fear	Provider-perspective qualitative study [34]: intensified immigration enforcement produced deportation fear extending to US-citizen children and wider networks, with healthcare avoidance, stress and anxiety.	Language-concordant study staff; omit immigration status from data collection where scientifically unnecessary; recruit through trusted community institutions; transparent, written data-protection statements.	Barrier: IV Solution: V
Study design	Loss to follow-up and participant untraceability	McKenzie et al. [35] (review of tracking marginalized populations); Cottler et al. [36] (96.6% follow-up); Worth et al. [37] (ED-based SUD study): multi-modal contact produced 3-, 6- and 12-month retention of 89%, 86% and 81%.	Obtain consent for multi-modal contact (phone, text, email, social media, home visit) at baseline; collect and refresh multiple collateral contacts at every visit; fund staff training, rapport-building and field-safety protocols.	III–IV (strongest operational evidence in this review)
Study design	Restrictive eligibility criteria and treatment-seeker bias	Ray et al. [38]; Rohn et al. [39]; Fein and Landman [40]; Lee et al. [41]: treatment seekers differ from non-seekers in drinking pattern, age, severity, impulsivity and liver function. General health-disparities recruitment frameworks have historically excluded individuals with active substance use.	Enroll both treatment-seeking and non-treatment-seeking participants; require explicit scientific justification for each exclusion criterion; report screening-to-enrolment yield and reasons for exclusion.	Barrier: III–IV Solution: V
Study design	Consent comprehension and health literacy	HANDLS [26]: all written materials were readability-tested using the Flesch–Kincaid scale and a video explaining study rationale was embedded in the informed consent process, described by investigators as integral to cohort recruitment and retention.	Readability-test all participant-facing materials; add a short explanatory video at consent; confirm understanding using teach-back before enrolment.	III/V † (component of a successful cohort; not independently evaluated)
System-level	Site gatekeeping, workforce shortage and limited clinician engagement	Balio et al. [42]: practice managers act as gatekeepers in primary care. Thørrisen et al. [43]: organizational time and resource constraints. Paradise and Wakeman [44]: addiction workforce shortages. TREAT coordinator survey [13]: the two most common requests were less time spent identifying eligible patients and greater physician involvement in recruitment.	Embed research staff within clinical workflow; automate EHR-based eligibility flagging; formalize clinician referral pathways; adopt multidisciplinary clinic models (ALD clinic [45]: 75% still active at 6 months with no abstinence requirement; 2023 retrospective cohort [46]: visit continuation 91.7% vs. 38.7% at 6 months and 90.6% vs. 25.8% at 12 months).	Barrier: IV Multidisciplinary model: III †
System-level	Chronic under-recruitment and inadequate recruitment planning	Watson et al. [47]; White and Hind [48]; Fogel [49]: recruitment shortfall is a long-standing cause of underpowered trials and publication bias; early planning and adequate remuneration are repeatedly identified as determinants of trial timelines.	Pre-specify recruitment projections, feasibility data and escalation or stopping triggers in the protocol; run an internal pilot with explicit progression criteria before full enrolment.	IV–V

Level of evidence (adapted from standard evidence hierarchies); I—Systematic review or meta-analysis of randomized controlled trials. II—Individual randomized controlled trial. III—Controlled observational study: prospective or retrospective cohort, or quasi-experimental design. IV—Cross-sectional survey, case series, qualitative study, or uncontrolled program evaluation. V—Expert opinion, mechanistic reasoning, or extrapolation without direct empirical test. † Indirect evidence: derived from non-AUD or mixed substance-use populations, or from studies in which the measured outcome was treatment engagement or continuation rather than research recruitment or retention. Applicability to AUD research is inferred and should be interpreted as suggestive. AUD, alcohol use disorder; ALD, alcohol-associated liver disease; CBT, cognitive behavioral therapy; CM, contingency management; ED, emergency department; EFT, episodic future thinking; EHR, electronic health record; RCT, randomized controlled trial; SDOH, social determinants of health; SUD, substance use disorder.

## Data Availability

No new data were created or analyzed in this study. Data sharing is not applicable to this article.

## Abbreviations

Alcohol use disorder (AUD), Cognitive behavioral therapy (CBT), Matching Alcoholism Treatment to Client Heterogeneity (MATCH), Emergency department (ED), Mental Health Parity and Addiction Equity Act (MHPAEA), Socioeconomic Status (SES), Social Determinants of Health (SDOH).
